# Editorial for Special Issue—Biomarkers of Renal Disease

**DOI:** 10.3390/ijms21218077

**Published:** 2020-10-29

**Authors:** Joaquín García-Estañ, Felix Vargas

**Affiliations:** 1 Departamento de Fisiologia, Facultad de Medicina, IMIB, Universidad de Murcia, 30120 Murcia, Spain; 2 Departamento de Fisiologia, Facultad de Medicina, Universidad de Granada, 18071 Granada, Spain

The National Institutes of Health (NIH) Biomarkers Definitions Group has defined a biomarker as “A characteristic that is objectively measured and evaluated as an indicator of normal biologic processes, pathogenic processes, or pharmacologic responses to a therapeutic intervention.” For acute or chronic kidney diseases, the ideal biomarker should, among others, show rapid and reliable changes with the progression of the disease and be highly sensitive and specific, be able to detect injury to the different segments of the nephron, and be rapidly and easily measurable. Creatinine, for instance, is not a good renal marker since acute injuries would not show changes in filtration rate until the progression of the disease allows its accumulation. Similarly, in chronic renal disease, the elevation in serum creatinine is a late indicator of the reduction in glomerular filtration. Other conventional biomarkers such as proteinuria, cell cylinders, and fractional excretion of sodium have shown lack of sensitivity and specificity for the early recognition of acute kidney injury; hence, leading to the need and the enormous interest surrounding the possibility of using other biomarkers with the ability to perform early detection, differential diagnosis, assessment prognostic, response to treatment, and functional recovery. In this Special Issue [1], we have published reviews or experimental papers showing significant advances in the field of renal biomarkers.

Regarding research articles, we have an interesting contribution by Groeneweg et al. [2] to the topic of rejection of a kidney graft. These authors demonstrate that the use of circulating long noncoding RNAs (lncRNAs) may be a suitable marker for vascular injury in that setting, specially LNC-EPHA6, a substance that has been found to relate to diabetic nephropathy. An additional paper [3] by Borštnar et al., working with microRNAs (miRNAs), concluded that six selected miRNAs (miR-29c, miR-126, miR-146a, miR-150, miR-155, and miR-223) were shown to be independent of kidney graft function, indicating their potential as biomarkers of associated kidney graft disease processes, but using serum uromodulin levels, which were also analyzed, depended entirely on kidney graft function and thus reflected functioning tubules rather than any specific kidney graft injury. In line with these studies, a good review by Guzzi et al. [4] followed the Preferred Reporting Items for Systematic Reviews and Meta-Analysis (PRISMA) guidelines to conclude that urinary C-X-C motif chemokine ligands were the most promising and frequently studied biomarkers for diagnosis and prediction of acute kidney allograft rejection. In the same field, the review by Quaglia and colleagues [5] explores new biomarkers of early and late graft dysfunction, which are very much needed in renal transplants to improve the management of complications and prolong graft survival. Thus, OMIC technology (all technologies aimed at detection of genes (genomics), mRNA (transriptomics), proteins (proteomics) and metabolites (metabolomics)) has allowed the identification of many candidate biomarkers, providing diagnostic and prognostic information at very early stages of pathological processes. Donor-derived cell-free DNA and extracellular vesicles are further promising tools. However, most of these biomarkers still need to be validated in multiple independent cohorts and standardized, and prospective studies are needed to assess whether introduction of these new sets of biomarkers into clinical practice could actually reduce the need for renal biopsy, integrate traditional tools, and ultimately improve graft survival compared to current management.

In an interesting study [6], Glazyrin and coworkers examined several machine learning algorithms linked to a full-proteomic approach, which were examined for the differential diagnosis of chronic kidney disease (CKD) of three origins, diabetic nephropathy, hypertension, and glomerulonephritis—three of the most common causes of CKD. While the group of hypertensive nephropathy could not be reliably separated according to plasma data, this group of hypertensive nephropathy was reliably separated from all other renal patients by urine proteome data. However, the analysis of the entire proteomics data of urine did not allow differentiating between the three diseases. Thus, it seems that the urine proteome, compared with the plasma proteome, is of much less importance. Clearly, this is an area of interest that will benefit from the incorporation of data technicians and proteomic analysts to these hospital services. Additional information came with the results shown in the article by Ahn et al. [7]. They used proteome analysis for the prediction of type 2 diabetic patients with or without renal dysfunction. In the results of these authors, it looks that several proteins (ACP2, CTSA, GM2A, MUC1, and SPARCL1) performed better than mucin-1 or albumin as predictors of direct kidney function in these diabetic patients with kidney impairment.

Tseng et al. [8], in the field of renal fibrosis, showed that histone deacetylase inhibition by trichostatin A significantly attenuated renal fibrosis through promoting an M1(proinflammatory) to M2 (anti-inflammatory) macrophage transition in obstructed kidneys, therefore alleviating the renal fibrosis in obstructed kidneys. However, it is first necessary to establish the role of M2 macrophages regarding its profibrotic or antifibrotic roles.

An important review by Provenzano and coworkers [9] has reported a framework for implementing biomarkers in observational and intervention studies. To that end, biomarkers are classified as either prognostic or predictive, the first type is used to identify the likelihood of a patient to develop an endpoint regardless of treatment, whereas the second type is used to determine whether the patient is likely to benefit from a specific treatment. Thus, the authors revise current biomarkers useful for chronic kidney patients, not only kidney biomarkers but also markers of oxidative stress, tissue remodeling, metabolism, and cardiac biomarkers, together with some important paragraphs on the role, either prognostic or predictive, of proteomics, metabolomics, and genomics. A final page on biomarkers in intervention studies should be of interest to clinical studies and those in the experimental phase of drug development.

The contribution by Petejova et al. [10] covered the pathophysiology of vancomycin and gentamicin nephrotoxicity. In particular, septic acute kidney injury (AKI) and the microRNAs involved in the pathophysiology of both syndromes and also the pathophysiology and potential biomarkers of septic and toxic acute kidney injury in septic patients was studied. In addition, five miRNAs (miR-15a-5p, miR-192-5p, miR-155-5p, miR-486-5p and miR-423-5p) specific to septic and toxic acute kidney injury in septic patients, treated by nephrotoxic antibiotic agents (vancomycin and gentamicin), were identified.

Partial or complete obstruction of the urinary tract is a common and challenging urological condition caused by a variety of conditions, eventually impairing renal function. Washino and coworkers [11] report that biomarkers of acute kidney injury are useful for the early detection and monitoring of kidney injury induced by upper urinary tract obstruction, including levels of neutrophil gelatinase-associated lipocalin (NGAL), monocyte chemotactic protein-1, kidney injury molecule 1, N-acetyl-b-D-glucosaminidase, and vanin-1 in the urine and serum NGAL and cystatin C concentrations.

In a review by the group of Vargas et al. [12], they focused on the role of four aminopeptidases in the control of blood pressure (BP) and renal function and their association with different cardiovascular and renal diseases. Beyond their role as therapeutic tools for BP control and renal diseases, they also explored their role as urinary biomarkers of renal injury in both acute and chronic renal nephropathies, including those induced by nephrotoxic agents, obesity, hypertension, or diabetes.

The review by Wołyniec et al. [13] has identified and analyzed several studies that have studied these markers after physical exercise, concluding that there is evidence that cystatin C is a better indicator of glomerular filtration rate (GFR) in athletes after exercise than creatinine. Additionally, serum and plasma NGAL are increased after prolonged exercise, but the level also depends on inflammation and hypoxia; therefore, it seems that in physical exercise, it is too sensitive for AKI diagnosis. It may, however, help to diagnose subclinical kidney injury, e.g., in rhabdomyolysis. Although urinary biomarkers are increased after many types of exercise, such as NGAL, KIM-1, cystatin-C, L-FABP and interleukin 18, their levels decrease rapidly after exercise; thus, the importance of this short-term increase in AKI biomarkers after exercise lacks a physiological explanation and it merits further studies that show their relation to kidney injury.

In the search for biomarkers of acute tubulointerstitial nephritis, Martinez-Valenzuela and coworkers [14] have summarized the available evidence on this topic, with a special focus on urinary cytokines and chemokines that may reflect kidney local inflammation. However, they conclude that to date, there is a lack of reliable non-invasive diagnostic and follow-up markers and that the gold standard for diagnosis is still kidney biopsy, which shows a pattern of tubulointerstitial leukocyte infiltrate.

Coca et al. have revised the [15] furosemide stress test as a low-cost, fast, safe, and easy-to-perform test to assess tubular integrity, to allow for risk stratification and accurate patient prognosis in the management of patients with kidney disease. However, the findings published so far regarding its clinical use provide insufficient evidence to recommend the generalized application of the test in daily clinical routine, and they recommend the need for standardization in the application of the test in order to facilitate the comparison of results.

Finally, Uchida et al. [16] write about the glomerulonephritis that often develops after the curing of an infection, such as the glomerulonephritis (GN) in children following streptococcal infections (poststreptococcal acute glomerulonephritis, PSAGN). Nephritis-associated plasmin receptor (NAPlr), isolated from the cytoplasmic fraction of group A streptococcus, has been shown to trap plasmin and maintain its activity and was originally considered as a nephritogenic protein for PSAGN. Indeed, NAPlr deposition and related plasmin activity have been observed to have an almost identical distribution in the glomeruli of early phase PSAGN patients at a high frequency. The authors conclude that the interactions among NAPlr, plasmin activity, and the streptococcal cysteine proteinase SpeB, and the association between these elements and complements or immune complexes, both in vitro and in vivo, should be investigated in future studies.

## References

[1] Biomarkers of Renal Disease. Special Issue. https://www.mdpi.com/journal/ijms/special_issues/Biomarkers_Renal_Diseases#published.

[2] Groeneweg K.E., Duijs J.M., Florijn B.W., van Kooten C., de Fijter J.W., van Zonneveld A.J., Reinders M.E., Bijkerk R. (2020). Circulating Long Noncoding RNA LNC-EPHA6 Associates with Acute Rejection after Kidney Transplantation. Int. J. Mol. Sci..

[3] Borštnar Š., Večerić-Haler Ž., Boštjančič E., Pipan Tkalec Ž., Kovač D., Lindič J., Kojc N. (2020). Uromodulin and microRNAs in Kidney Transplantation—Association with Kidney Graft Function. Int. J. Mol. Sci..

[4] Guzzi F., Cirillo L., Buti E., Becherucci F., Errichiello C., Roperto R.M., Hunter J.P., Romagnani P. (2020). Urinary Biomarkers for Diagnosis and Prediction of Acute Kidney Allograft Rejection: A Systematic Review. Int. J. Mol. Sci..

[5] Quaglia M., Merlotti G., Guglielmetti G., Castellano G., Cantaluppi V. (2020). Recent Advances on Biomarkers of Early and Late Kidney Graft Dysfunction. Int. J. Mol. Sci..

[6] Glazyrin Y.E., Veprintsev D.V., Ler I.A., Rossovskaya M.L., Varygina S.A., Glizer S.L., Zamay T.N., Petrova M.M., Minic Z., Berezovski M.V. (2020). Proteomics-Based Machine Learning Approach as an Alternative to Conventional Biomarkers for Differential Diagnosis of Chronic Kidney Diseases. Int. J. Mol. Sci..

[7] Ahn H.-S., Kim J.H., Jeong H., Yu J., Yeom J., Song S.H., Kim S.S., Kim I.J., Kim K. (2020). Differential Urinary Proteome Analysis for Predicting Prognosis in Type 2 Diabetes Patients with and without Renal Dysfunction. Int. J. Mol. Sci..

[8] Tseng W.-C., Tsai M.-T., Chen N.-J., Tarng D.-C. (2020). Trichostatin A Alleviates Renal Interstitial Fibrosis through Modulation of the M2 Macrophage Subpopulation. Int. J. Mol. Sci..

[9] Provenzano M., Rotundo S., Chiodini P., Gagliardi I., Michael A., Angotti E., Borrelli S., Serra R., Foti D., De Sarro G. (2020). Contribution of Predictive and Prognostic Biomarkers to Clinical Research on Chronic Kidney Disease. Int. J. Mol. Sci..

[10] Petejova N., Martinek A., Zadrazil J., Kanova M., Klementa V., Sigutova R., Kacirova I., Hrabovsky V., Svagera Z., Stejskal D. (2020). Acute Kidney Injury in Septic Patients Treated by Selected Nephrotoxic Antibiotic Agents—Pathophysiology and Biomarkers—A Review. Int. J. Mol. Sci..

[11] Washino S., Hosohata K., Miyagawa T. (2020). Roles Played by Biomarkers of Kidney Injury in Patients with Upper Urinary Tract Obstruction. Int. J. Mol. Sci..

[12] Vargas F., Wangesteen R., Rodríguez-Gómez I., García-Estañ J. (2020). Aminopeptidases in Cardiovascular and Renal Function. Role as Predictive Renal Injury Biomarkers. Int. J. Mol. Sci..

[13] Wołyniec W., Ratkowski W., Renke J., Renke M. (2020). Changes in Novel AKI Biomarkers after Exercise. A Systematic Review. Int. J. Mol. Sci..

[14] Martinez Valenzuela L., Draibe J., Fulladosa X., Torras J. (2020). New Biomarkers in Acute Tubulointerstitial Nephritis: A Novel Approach to a Classic Condition. Int. J. Mol. Sci..

[15] Coca A., Aller C., Reinaldo Sánchez J., Valencia A.L., Bustamante-Munguira E., Bustamante-Munguira J. (2020). Role of the Furosemide Stress Test in Renal Injury Prognosis. Int. J. Mol. Sci..

[16] Uchida T., Oda T. (2020). Glomerular Deposition of Nephritis-Associated Plasmin Receptor (NAPlr) and Related Plasmin Activity: Key Diagnostic Biomarkers of Bacterial Infection-related Glomerulonephritis. Int. J. Mol. Sci..

